# Supplementary material for: Hybrid Models and Biological Model Reduction with PyDSTool
Source: PLoS Comput Biol. 2012 Aug 9;8(8):e1002628. doi: 10.1371/journal.pcbi.1002628 (PMC3415397; doi:10.1371/journal.pcbi.1002628)
Supplement: Text S4 — Complete source code for the PyDSTool package (version 0.88.120504). Includes API documentation and help files linking to web pages. This file is identical to the current public release on Sourceforge.net. (ZIP) [file pcbi.1002628.s004.zip › PyDSTool/html/PyDSTool.integrator'-pysrc.html]

xml version="1.0" encoding="ascii"?


PyDSTool.integrator'


| Home | Trees | Indices | Help | PyDSTool |
| --- | --- | --- | --- | --- |

Package PyDSTool ::
Module integrator'

[hide private]

[frames] | no frames]

# Source Code for Module PyDSTool.integrator'

```
  1  """Basic integrator interface class
 
  2     Erik Sherwood, September 2006
 
  3  """ 
  4  
 
  5  from errors import PyDSTool_InitError as InitError 
  6  from errors import PyDSTool_ClearError as ClearError 
  7  from common import _all_int, _real_types, \
 
  8       verify_intbool, verify_pos, verify_nonneg, verify_values 
  9  from numpy import isinf, Inf 
 10  import operator 
 11  import sys 
 12  
 
 13  # Need to add checks for when tolerances that should be nonneg are
 
 14  # less than 0
 


15 -class integrator:


16 -    def __init__(self, rhs='default_name', phaseDim=0, paramDim=0, nAux=0,
 
 17                   nEvents=0, nExtInputs=0, hasJac=0, hasJacP=0, hasMass=0,
 
 18                   extraSpace=0, defaultBound=1e8):


19  
 
 20          # Internal state variables
 
 21          self.initBasic = False 
 22          self.initIntegrate = False 
 23          self.initEvents = False 
 24          self.initExtInputs = False 
 25          self.setParams = False 
 26          self.numRuns = 0 
 27          self.numContinues = 0 
 28          self.canContine = False 
 29  
 
 30          # Run output
 
 31          self.times = [] 
 32          self.points = [] 
 33          self.auxPoints = [] 
 34          self.eventTimes = [] 
 35          self.eventPoints = [] 
 36          self.errors = [] 
 37          self.stats = [] 
 38          self.step = [] 
 39  
 
 40          self.lastPoint = [] 
 41          self.lastTime = [] 
 42  
 
 43          # Run parameters
 
 44          self.ic = []; self.params = [] 
 45          self.t0 = []; self.tend = []; self.gt0 = [] 
 46          self.refine = [] 
 47          self.upperBounds = [] 
 48          self.lowerBounds = [] 
 49          self.defaultBound = abs(float(defaultBound)) 
 50  
 
 51          # Event variables
 
 52          self.maxevtpts = [] 
 53          self.eventActive = []; self.eventDir = []; self.eventTerm = [] 
 54          self.eventInt = []; self.eventDelay=[]; self.eventTol=[] 
 55          self.maxbisect = [] 
 56  
 
 57          # Integ variables
 
 58          self.maxpts = []; self.rtol = []; self.atol = [] 
 59  
 
 60          # Specific to integrator
 
 61          self.hinit = []; self.hmax = []; self.verbose = [] 
 62          self.specTimes = [] 
 63          self.direction = 0 
 64  
 
 65          self.checkAux = 0 
 66          self.calcSpecTimes = 0 
 67  
 
 68          self.checkBasic(rhs=rhs, phaseDim=phaseDim, paramDim=paramDim,
 
 69                             nAux=nAux, nEvents=nEvents, nExtInputs=nExtInputs,
 
 70                             hasJac=hasJac, hasJacP=hasJacP, hasMass=hasMass,
 
 71                             extraSpace=extraSpace) 
 72  
 
 73          self.rhs = rhs 
 74          self.phaseDim = phaseDim 
 75          self.paramDim = paramDim 
 76          self.extraSpace = extraSpace 
 77          self.nAux = nAux 
 78          self.nEvents = nEvents 
 79          self.nExtInputs = nExtInputs 
 80  
 
 81          self.hasJac = int(hasJac) 
 82          self.hasJacP = int(hasJacP) 
 83          self.hasMass = int(hasMass)

 84  
 
 85          # At this point, we expect the child class to set the self._integ field
 
 86          # and then call the initBasic method of the shared library.
 
 87          # The child class sets self.initBasic to True
 
 88  
 
 89  
 


90 -    def __del__(self):


91          try: 
 92              self._integMod.CleanUp() 
 93          except: 
 94              pass

 95  
 
 96  
 


97 -    def checkBasic(self, rhs, phaseDim, paramDim, nAux, nEvents, nExtInputs,
 
 98                     hasJac, hasJacP, hasMass, extraSpace):


99          # Check that inputs to this function are correct
 
100          try: 
101              if not isinstance(rhs, str): 
102                  raise TypeError("right hand side rhs must be a string") 
103              verify_nonneg('phaseDim', phaseDim, _all_int) 
104              verify_nonneg('paramDim', paramDim, _all_int) 
105              verify_intbool('hasJac', hasJac) 
106              verify_intbool('hasJacP', hasJacP) 
107              verify_intbool('hasMass', hasMass) 
108              verify_nonneg('nAux', nAux, _all_int) 
109              verify_nonneg('nEvents', nEvents, _all_int) 
110              verify_nonneg('nExtInputs', nExtInputs, _all_int) 
111              verify_nonneg('extraSpace', extraSpace, _all_int) 
112          except: 
113              print sys.exc_info()[0], sys.exc_info()[1] 
114              raise InitError('Integrator initialization failed!')

115  
 
116  
 


117 -    def setEvents(self, maxevtpts=1000, eventActive=[], eventDir=[], eventTerm=[],
 
118                    eventInt=0.005, eventDelay=1e-4, eventTol=1e-6, maxbisect=100,
 
119                    eventNearCoef=1000):


120  
 
121          if not self.initBasic: 
122              raise InitError('You must initialize the integrator before setting events. (initBasic)') 
123  
 
124          if self.initEvents: 
125              raise InitError('You must clear events before setting them. Use clearEvents()') 
126  
 
127          # Currently we will not raise an error, but instead ignore setting events
 
128          # if nEvents is zero. Just set to some default values and pass to the
 
129          # shared library
 
130  
 
131          if self.nEvents <= 0: 
132              maxevtpts = 0 
133              eventActive = [] 
134              eventDir = [] 
135              eventTerm = [] 
136              eventInt = 0.005 
137              eventDelay = 1e-4 
138              eventTol = 1e-6 
139              maxbisect = 100 
140              eventNearCoef=1000 
141  
 
142          self.checkEvents(maxevtpts, eventActive, eventDir, eventTerm,
 
143                           eventInt, eventDelay, eventTol, maxbisect, eventNearCoef) 
144          self.maxevtpts = maxevtpts 
145  
 
146          if isinstance(eventActive, list): 
147              self.eventActive = [int(ea) for ea in eventActive] 
148          else: 
149              self.eventActive = [int(eventActive)]*self.nEvents 
150  
 
151          if isinstance(eventDir, list): 
152              self.eventDir = eventDir 
153          else: 
154              self.eventDir = [eventDir]*self.nEvents 
155  
 
156          if isinstance(eventTerm, list): 
157              self.eventTerm = [int(et) for et in eventTerm] 
158          else: 
159              self.eventTerm = [int(eventTerm)]*self.nEvents 
160  
 
161          if isinstance(maxbisect, list): 
162              self.maxbisect = maxbisect 
163          else: 
164              self.maxbisect = [maxbisect]*self.nEvents 
165  
 
166          if isinstance(eventInt, list): 
167              self.eventInt = [float(ei) for ei in eventInt] 
168          else: 
169              self.eventInt = [float(eventInt)]*self.nEvents 
170  
 
171          if isinstance(eventDelay, list): 
172              self.eventDelay = [float(ed) for ed in eventDelay] 
173          else: 
174              self.eventDelay = [float(eventDelay)]*self.nEvents 
175  
 
176          if isinstance(eventTol, list): 
177              self.eventTol = [float(et) for et in eventTol] 
178          else: 
179              self.eventTol = [float(eventTol)]*self.nEvents 
180  
 
181          self.eventNearCoef = eventNearCoef 
182  
 
183          retval = self._integMod.InitEvents(self.maxevtpts, self.eventActive,
 
184                                             self.eventDir, self.eventTerm,
 
185                                             self.eventInt, self.eventDelay,
 
186                                             self.eventTol, self.maxbisect,
 
187                                             self.eventNearCoef) 
188  
 
189          if retval[0] != 1: 
190              raise InitError('InitEvents call failed!') 
191  
 
192          self.canContinue = False 
193          self.initEvents = True

194  
 
195  
 


196 -    def clearEvents(self):


197          if not self.initBasic: 
198              raise ClearError('You must initialize the integrator before clearing events.') 
199          if self.initEvents: 
200              if self._integMod.ClearEvents()[0] != 1: 
201                  raise ClearError('ClearEvents call failed!') 
202              self.canContinue = False 
203              self.initEvents = False

204  
 
205  
 


206 -    def checkEvents(self, maxevtpts, eventActive, eventDir, eventTerm,
 
207                      eventInt, eventDelay, eventTol, maxbisect, eventNearCoef):


208  
 
209          if not self.initBasic: 
210              raise InitError('You must initialize the integrator before ' + \
 
211                              'checking events. (initBasic)') 
212  
 
213          verify_nonneg('maxevtpts', maxevtpts, _all_int) 
214  
 
215          list_len_val = (self.nEvents, 'nEvents') 
216  
 
217          verify_intbool('eventActive', eventActive,
 
218                        list_ok=True, list_len=list_len_val) 
219  
 
220          verify_values('eventDir', eventDir, [-1, 0, 1], list_ok=True,
 
221                        list_len=list_len_val) 
222  
 
223          verify_intbool('eventTerm', eventTerm,
 
224                         list_ok=True, list_len=list_len_val) 
225  
 
226          verify_nonneg('eventInt', eventInt, _real_types,
 
227                        list_ok=True, list_len=list_len_val) 
228  
 
229          verify_nonneg('eventDelay', eventDelay, _real_types,
 
230                        list_ok=True, list_len=(self.nEvents, 'nEvents')) 
231  
 
232          verify_pos('eventTol', eventTol, _real_types,
 
233                     list_ok=True, list_len=list_len_val) 
234  
 
235          verify_nonneg('maxbisect', maxbisect, _all_int,
 
236                        list_ok=True, list_len=list_len_val) 
237  
 
238          verify_pos('eventNearCoef', eventNearCoef, _real_types)

239  
 
240  
 


241 -    def setInteg(self, maxpts=100000, rtol=1e-9, atol=1e-12):


242          if not self.initBasic: 
243              raise InitError('You must initialize the integrator before setting integ. (initBasic)') 
244  
 
245          if self.initIntegrate: 
246              raise InitError('You must clear integ before setting it. Use clearInteg()') 
247  
 
248          self.checkInteg(maxpts, rtol, atol) 
249          self.maxpts = maxpts 
250  
 
251          if isinstance(rtol, list): 
252              self.rtol = rtol 
253          else: 
254              self.rtol = [rtol]*self.phaseDim 
255  
 
256          if isinstance(atol, list): 
257              self.atol = atol 
258          else: 
259              self.atol = [atol]*self.phaseDim 
260  
 
261          if self._integMod.InitInteg(self.maxpts, self.atol, self.rtol)[0] != 1: 
262              raise InitError('InitInteg call failed!') 
263  
 
264          self.canContinue = False 
265          self.initIntegrate = True

266  
 
267  
 


268 -    def clearInteg(self):


269          if not self.initBasic: 
270              raise ClearError('You must initialize the integrator before clearing events.') 
271          if self.initIntegrate: 
272              if self._integMod.ClearInteg()[0] != 1: 
273                  raise ClearError('ClearInteg call failed!') 
274              self.canContinue = False 
275              self.initIntegrate = False

276  
 
277  
 


278 -    def checkInteg(self, maxpts, rtol, atol):


279          if not self.initBasic: 
280              raise InitError('You must initialize the integrator before checking integ. (initBasic)') 
281  
 
282          verify_pos('maxpts', maxpts, _all_int) 
283  
 
284          list_len_val = (self.phaseDim, 'phaseDim') 
285          verify_pos('rtol', rtol, _real_types,
 
286                     list_ok=True, list_len=list_len_val) 
287          verify_pos('atol', atol, _real_types,
 
288                     list_ok=True, list_len=list_len_val)

289  
 
290  
 


291 -    def setExtInputs(self, doCheck, extInputVals, extInputTimes):


292          if not self.initBasic: 
293              raise InitError('You must initialize the integrator before setting external Inputs. (initBasic)') 
294  
 
295          if self.initExtInputs: 
296              raise InitError('You must clear extInputs before setting it. Use clearInteg()') 
297  
 
298          if self.nExtInputs > 0: 
299              if doCheck: 
300                  self.checkExtInputs(extInputVals, extInputTimes) 
301              self.extInputLens = [] 
302              for x in range(self.nExtInputs): 
303                  self.extInputLens.append(len(extInputTimes[x])) 
304  
 
305              IVals = extInputVals[0] 
306              ITimes = extInputTimes[0] 
307              for x in range(self.nExtInputs - 1): 
308                  IVals += extInputVals[x+1] 
309                  ITimes += extInputTimes[x+1] 
310  
 
311              self.extInputVals = extInputVals 
312              self.extInputTimes = extInputTimes 
313  
 
314              if self._integMod.InitExtInputs(self.nExtInputs,
 
315                             self.extInputLens, IVals, ITimes)[0] != 1: 
316                  raise InitError('InitExtInputs call failed!') 
317  
 
318              self.canContinue = False 
319              self.initExtInputs = True

320  
 
321  
 


322 -    def clearExtInputs(self):


323          if not self.initBasic: 
324              raise ClearError('You must initialize the integrator before clearing external inputs.') 
325          if self.nExtInputs <= 0: 
326              self.extInputLens = [] 
327              self.extInputVals = [] 
328              self.extInputTimes = [] 
329              self.initExtInputs = False 
330          elif self.initExtInputs: 
331              if self._integMod.ClearExtInputs()[0] != 1: 
332                  raise ClearError('ClearExtInputs call failed!') 
333              self.extInputLens = [] 
334              self.extInputVals = [] 
335              self.extInputTimes = [] 
336              self.canContinue = False 
337              self.initExtInputs = False

338  
 
339  
 


340 -    def checkExtInputs(self, extInputVals, extInputTimes):


341          if not self.initBasic: 
342              raise InitError('You must initialize the integrator before checking external inputs. (initBasic)') 
343  
 
344          if not isinstance(extInputVals, list): 
345              raise TypeError("extInputVals must be list.") 
346          if len(extInputVals) != self.nExtInputs: 
347              raise ValueError("length of extInputVals must match nExtInputs") 
348          for x in range(self.nExtInputs): 
349              for y in range(len(extInputVals[x])): 
350                  if not isinstance(extInputVals[x][y], _real_types): 
351                      raise TypeError("extInputVals entries must be real values") 
352  
 
353          for x in range(self.nExtInputs): 
354              for y in range(len(extInputTimes[x])): 
355                  if not isinstance(extInputVals[x][y], _real_types): 
356                      raise TypeError("extInputTimes entries must be real values") 
357              if len(extInputTimes[x]) > 1: 
358                  # Check orientation
 
359                  orientation = extInputTimes[x][-1] - extInputTimes[x][0] 
360                  if orientation == 0: 
361                      raise ValueError("Each extInputTime must be distinct; first and last times are identical with len > 1") 
362                  if orientation < 0: 
363                      for y in range(len(extInputTimes[x])-1): 
364                          if extInputTimes[x][y] <= extInputTimes[x][y+1]: 
365                              raise ValueError("extInputTimes must be ordered consistently") 
366                  if orientation > 0: 
367                      for y in range(len(extInputTimes[x])-1): 
368                          if extInputTimes[x][y] >= extInputTimes[x][y+1]: 
369                              print x, y, extInputTimes[x][y],extInputTimes[x][y+1] 
370                              raise ValueError("extInputTimes must be ordered consistently")

371  
 
372  
 


373 -    def setRunParams(self, ic=[], params=[], t0=[], tend=[], gt0=[], refine=0,
 
374                       specTimes=[], bounds=[]):


375          if not self.initBasic: 
376              raise InitError('You must initialize the integrator before setting params. (initBasic)') 
377  
 
378          #if self.initParams:
 
379          #    raise InitError('You must clear params before setting them. Use clearParams()')
 
380          self.checkRunParams(ic, params, t0, tend, gt0, refine, specTimes,
 
381                                 bounds) 
382          self.ic = ic 
383          self.params = params 
384          self.t0 = float(t0) 
385          self.tend = float(tend) 
386          self.gt0 = float(gt0) 
387          self.refine = int(refine) 
388          self.specTimes = list(specTimes) 
389  
 
390          if self.t0 < self.tend: 
391              self.direction = 1 
392          else: 
393              self.direction = -1 
394  
 
395          # Set bounds
 
396          if bounds != []: 
397              self.upperBounds = bounds[1] 
398              self.lowerBounds = bounds[0] 
399              for i in range(self.phaseDim + self.paramDim): 
400                  if isinf(self.upperBounds[i]) and self.upperBounds[i] > 0: 
401                      self.upperBounds[i] = abs(float(self.defaultBound)) 
402                  elif isinf(self.upperBounds[i]) and self.upperBounds[i] < 0: 
403                      self.upperBounds[i] = -abs(float(self.defaultBound)) 
404  
 
405                  if isinf(self.lowerBounds[i]) and self.lowerBounds[i] > 0: 
406                      self.lowerBounds[i] = abs(float(self.defaultBound)) 
407                  elif isinf(self.lowerBounds[i]) and self.lowerBounds[i] < 0: 
408                      self.lowerBounds[i] = -abs(float(self.defaultBound)) 
409          else: 
410              self.upperBounds = [abs(float(self.defaultBound))] * \
 
411                  (self.phaseDim + self.paramDim) 
412              self.lowerBounds = [-abs(float(self.defaultBound))] * \
 
413                  (self.phaseDim + self.paramDim) 
414  
 
415          retval = self._integMod.SetRunParameters(self.ic, self.params,
 
416                               self.gt0, self.t0, self.tend, self.refine,
 
417                               len(self.specTimes), self.specTimes,
 
418                               self.upperBounds, self.lowerBounds) 
419  
 
420          if retval[0] != 1: 
421              raise InitError('SetRunParameters call failed!') 
422  
 
423          self.canContinue = False 
424          self.setParams = True

425  
 
426  
 


427 -    def clearRunParams(self):


428          if not self.initBasic: 
429              raise ClearError('You must initialize the integrator before clearing params.') 
430          if self.setParams: 
431              if self._integMod.ClearParams()[0] != 1: 
432                  raise ClearError('ClearParams call failed!') 
433              self.canContinue = False 
434              self.setParams = False

435  
 
436  
 


437 -    def checkRunParams(self, ic, params, t0, tend, gt0, refine, specTimes,
 
438                         bounds):


439          if not self.initBasic: 
440              raise InitError('You must initialize the integrator before checking run params. (initBasic)') 
441  
 
442          if not isinstance(ic, list): 
443              raise TypeError("ic must be list") 
444          if len(ic) != self.phaseDim: 
445              print "IC length %i didn't match phaseDim %i"%(len(ic), self.phaseDim), ic 
446              raise ValueError('ic must have length equal to phaseDim') 
447          for x in ic: 
448              if not isinstance(x, _real_types): 
449                  raise TypeError("ic entries must be real values") 
450  
 
451          if not isinstance(params, list): 
452              raise TypeError("params must be list") 
453          if len(params) != self.paramDim: 
454              raise ValueError("params must have length equal to phaseDim") 
455          if len(params) > 0: 
456              for x in params: 
457                  if not isinstance(x, _real_types): 
458                      raise TypeError("params entries must be real values") 
459  
 
460          verify_nonneg('refine', refine, _all_int) 
461  
 
462          if not isinstance(t0, _real_types): 
463              raise TypeError("t0 must be real valued") 
464          if not isinstance(tend, _real_types): 
465              raise TypeError("tend must be real valued") 
466          if t0 == tend: 
467              raise ValueError("t0 must differ from tend") 
468          if t0 < tend: 
469              direction = 1 
470          else: 
471              direction = -1 
472  
 
473          try: 
474              specTimes = list(specTimes) 
475          except: 
476              raise TypeError("specTimes must be a sequence type") 
477          if len(specTimes) > 0: 
478              if not isinstance(specTimes[0], _real_types): 
479                  raise TypeError("specTimes entries must be real valued") 
480              if direction == 1: 
481                  if specTimes[0] < t0 or specTimes[0] > tend: 
482                      raise ValueError("specTimes entries must be within [%.8f,%.8f]"%(t0,tend)) 
483              else: 
484                  if specTimes[0] > t0 or specTimes[0] < tend: 
485                      raise ValueError("specTimes entries must be within [%.8f,%.8f]"%(tend,t0)) 
486  
 
487          if len(specTimes) > 1: 
488              for x in range(len(specTimes)-1): 
489                  if not isinstance(specTimes[x], _real_types): 
490                      raise TypeError("specTimes entries must be real valued") 
491              if direction == 1: 
492                  if specTimes[x] < t0 or specTimes[x] > tend: 
493                      raise ValueError("specTimes entries must be within [%.8f,%.8f]"%(t0,tend)) 
494                  if specTimes[x] > specTimes[x+1]: 
495                      raise ValueError("specTimes must be non-decreasing") 
496              else: 
497                  if specTimes[x] > t0 or specTimes[x] < tend: 
498                      raise ValueError("specTimes entries must be within [%.8f,%.8f]"%(tend,t0)) 
499                  if specTimes[x] < specTimes[x+1]: 
500                      raise ValueError("specTimes must be non-increasing") 
501  
 
502  
 
503          # Check the parameter, phase space bounds
 
504          if not isinstance(bounds, list): 
505              raise TypeError("bounds must be list") 
506          if bounds != []: 
507              if len(bounds) != 2: 
508                  raise TypeError("non-empty bounds must be a 2-list") 
509              for i in range(2): 
510                  if type(bounds[i]) is not list: 
511                      raise TypeError("non-empty bounds must be a 2-list") 
512                  if len(bounds[i]) != self.phaseDim + self.paramDim: 
513                      raise ValueError("bounds have incorrect size") 
514                  for j in range(self.phaseDim + self.paramDim): 
515                      if not isinstance(bounds[i][j], _real_types): 
516                          raise TypeError("bounds entries must be real valued")

517  
 
518  
 


519 -    def setContParams(self, tend, params, calcSpecTimes, verbose,
 
520                        extInputChanged, extInputVals, extInputTimes, bounds):


521          if self.direction > 0: 
522              if tend < self.tend: 
523                  raise ValueError("new tend must be > old tend") 
524          if self.direction < 0: 
525              if tend > self.tend: 
526                  raise ValueError("new tend must be < old tend") 
527  
 
528          if not isinstance(params, list): 
529              raise TypeError("params must be list") 
530          if params != []: 
531              if len(params) != self.paramDim: 
532                  raise ValueError("params must have length equal to phaseDim") 
533              if len(params) > 0: 
534                  for x in params: 
535                      if not isinstance(x, _real_types): 
536                          raise TypeError("params entries must be real valued") 
537  
 
538          if calcSpecTimes not in [0,1]: 
539              raise TypeError("calcSpecTimes must be 0 or 1") 
540          if calcSpecTimes == 1 and len(self.specTimes) <= 0: 
541              raise ValueError("calcSpecTimes cannot be 1 if specTimes is empty") 
542  
 
543          if verbose not in [0,1]: 
544              raise TypeError("verbose must be 0 or 1") 
545  
 
546          if extInputChanged: 
547              if self.nExtInputs <= 0: 
548                  raise ValueError("Cannot change extInputs if nExtInputs is 0") 
549              if self.initExtInputs: 
550                  self.checkExtInputs(extInputVals, extInputTimes) 
551                  self.clearExtInputs() 
552                  self.setExtInputs(False, extInputVals, extInputTimes) 
553              else: 
554                  self.setExtInputs(True, extInputVals, extInputTimes) 
555  
 
556          # Check and set the parameter, phase space bounds
 
557          if not isinstance(bounds, list): 
558              raise TypeError("bounds must be list") 
559          if bounds != []: 
560              if len(bounds) != 2: 
561                  raise TypeError("non-empty bounds must be a 2-list") 
562              for i in range(2): 
563                  if not isinstance(bounds[i], list): 
564                      raise TypeError("non-empty bounds must be a 2-list") 
565                  if len(bounds[i]) != self.phaseDim + self.paramDim: 
566                      raise ValueError("bounds have incorrect size") 
567                  for j in range(self.phaseDim + self.paramDim): 
568                      if not isinstance(bounds[i][j], _real_types): 
569                          raise TypeError("entries in bounds must be real valued") 
570  
 
571              self.upperBounds = bounds[0] 
572              self.lowerBounds = bounds[1] 
573              for i in range(self.phaseDim + self.paramDim): 
574                  if self.upperBounds[i] == Inf: 
575                      self.upperBounds[i] = abs(float(self.defaultBound)) 
576                  elif self.upperBounds[i] == -Inf: 
577                      self.upperBounds[i] = -abs(float(self.defaultBound)) 
578  
 
579                  if self.lowerBounds[i] == Inf: 
580                      self.lowerBounds[i] = abs(float(self.defaultBound)) 
581                  elif self.lowerBounds[i] == -Inf: 
582                      self.lowerBounds[i] = -abs(float(self.defaultBound)) 
583  
 
584          # in case params blank, leave alone
 
585          if params != []: 
586              self.params = params 
587          self.tend = tend 
588  
 
589          retval = self._integMod.SetContParameters(self.tend, self.params,
 
590                                           self.upperBounds, self.lowerBounds) 
591          if retval[0] != 1: 
592              raise InitError('Call to SetContParams failed!')

593  
 


594 -    def clearAll(self):


595          if not self.initBasic: 
596              raise InitError('You must initialize the integrator before clearing') 
597          self.clearRunParams() 
598          self.clearEvents() 
599          self.clearInteg() 
600          self.clearExtInputs()

601  
 


602 -    def Run(*args):


603          if self.__class__==integrator: 
604              raise NotImplementedError("Call Run on a concrete subclass")

605  
 


606 -    def Continue(*args):


607          if self.__class__==integrator: 
608              raise NotImplementedError("Call Continue on a concrete subclass")

609  
 


610 -    def Reset(self):


611          if not self.initBasic: 
612              raise InitError('You must initialize the integrator before resetting') 
613          self._integMod.Reset()

614          # What to do now?
 
615  
 


616 -    def Rhs(self, time, x, p):


617          if self.initBasic: 
618              if self.nExtInputs > 0 and not self.initExtInputs: 
619                  return None 
620              else: 
621                  return self._integMod.Vfield(time, x, p) 
622          return None

623  
 


624 -    def Jacobian(self, time, x, p):


625          if self.initBasic: 
626              if self.nExtInputs > 0 and not self.initExtInputs: 
627                  return None 
628              if self.hasJac: 
629                  return self._integMod.Jacobian(time, x, p) 
630          return None

631  
 


632 -    def JacobianP(self, time, x, p):


633          if self.initBasic: 
634              if self.nExtInputs > 0 and not self.initExtInputs: 
635                  return None 
636              if self.hasJacP: 
637                  return self._integMod.JacobianP(time, x, p) 
638          return None

639  
 


640 -    def MassMatrix(self, time, x, p):


641          if self.initBasic: 
642              if self.nExtInputs > 0 and not self.initExtInputs: 
643                  return None 
644              if self.hasMass: 
645                  return self._integMod.MassMatrix(time, x, p) 
646          return None

647  
 


648 -    def AuxFunc(self, time, x, p):


649          if self.initBasic: 
650              if self.nExtInputs > 0 and not self.initExtInputs: 
651                  return None 
652              if self.nAux > 0: 
653                  return self._integMod.AuxFunc(time, x, p) 
654          return None

655
```

  


| Home | Trees | Indices | Help | PyDSTool |
| --- | --- | --- | --- | --- |

|  |  |
| --- | --- |
| Generated by Epydoc 3.0.1 on Fri May 4 15:24:23 2012 | http://epydoc.sourceforge.net |
